# Efficacy of ceftaroline and rifampin, alone or combined, in a rat model of methicillin-resistant *Staphylococcus epidermidis* osteomyelitis without implant

**DOI:** 10.1128/spectrum.00153-23

**Published:** 2023-10-04

**Authors:** S. Albac, N. Anzala, D. Bonnot, H. Mirfendereski, P. Chavanet, D. Croisier

**Affiliations:** 1 Vivexia, Dijon, France; 2 Université de Poitiers, Poitiers, France; 3 Centre Hospitalier Universitaire de Poitiers, Poitiers, France; 4 Département d’Infectiologie, Centre Hospitalier Universitaire, Dijon, France; Hartford Hospital, Hartford, Connecticut, USA

**Keywords:** ceftaroline, bone penetration, MRSE, osteitis, rifampin, preclinical model, pharmacokinetics

## Abstract

The *in vivo* efficacy of ceftaroline and rifampin alone or in combination was investigated in an experimental rat model of methicillin-resistant *Staphylococcus epidermidis* (MRSE) osteomyelitis. After a 3-day treatment, the mean tibial bacterial load was significantly reduced in animals receiving rifampin alone or combined with ceftaroline (1.89 and 1.68 log_10_ CFU/g, respectively, *P* < 0.0001), whereas ceftaroline alone did not show any significant reduction at this early stage. After a 7-day treatment, the mean tibial bacterial load was reduced for ceftaroline alone compared to control animals (3.6 log_10_ CFU/g versus 4.4 log_10_ CFU/g, respectively, *P* < 0.05) and reached the limit of detection in almost all animals receiving rifampin alone or combined with ceftaroline (*P* < 0.0001). Finally, after a 14-day treatment the efficacy of ceftaroline alone was enhanced (*P* < 0.0001), and no MRSE was recovered from the tibias of animals receiving rifampin alone or combined with ceftaroline. No emergence of rifampin resistance was observed, regardless of the treatment group (monotherapy or combined therapy) or the timepoint of interest. Ceftaroline administered with rifampin could represent a valuable therapeutic option in the management of MRSE osteomyelitis.

**IMPORTANCE:**

Methicillin-resistant *Staphylococcus epidermidis* (MRSE) contributes to a high percentage of orthopedic infections, and their treatment represents a huge challenge. The present study aimed to evaluate the efficacy of ceftaroline alone or combined with rifampin in a rat MRSE osteomyelitis model and the bone penetration of ceftaroline. A ceftaroline monotherapy showed a significant bacterial reduction in infected bones after a 7-day period of treatment. The combination ceftaroline plus rifampin leveraged rifampin’s bactericidal activity, shortening the duration of positive culture in infected animals. These results suggest that ceftaroline and rifampin combination therapy could represent a valuable therapeutic option for human MRSE osteomyelitis and deserves further preclinical and clinical evaluation.

## INTRODUCTION

Osteomyelitis is an inflammatory bone disease caused by the presence of an infecting microorganism and leading to progressive bone destruction and loss. Approximately 50% to 60% of serious diabetic foot infections (DFIs) progress to osteomyelitis; 10% to 30% of these DFIs require lower extremity amputation (1, 2). The most common causative species are the usually commensal staphylococci, with *Staphylococcus aureus* and Coagulase-Negative Staphylococci (CoNS) being responsible for over half of osteomyelitis cases (3, 4).

Despite CoNS being considered as microorganisms with poor virulence, clinical studies with microbiological examination of bone samples have shown isolation rates of CoNS between 10% and 50% (5). CoNS are also isolated in around 25% of diabetic foot associated osteomyelitis (6). These findings support the idea that CoNS are true pathogens in such cases. Also, methicillin-resistant *S. epidermidis* (MRSE) strains are by far the most prevalent CoNS as they represent 60% to 70% of clinically recovered isolates in orthopedic infections (7, 8).

Treatment of bacterial osteomyelitis represents a huge challenge to public health because of the widespread antimicrobial resistance and the difficult penetration of antibiotics into the infection site. Long-term antibiotic therapies are required, in association with surgical debridement of necrotic infected tissues (8 – 10). Vancomycin remains the treatment of choice of MRSE osteomyelitis, but the long period of therapy is often accompanied by intolerance and for isolates having an MIC ≥2 mg/L, the use of an alternative therapeutic agent may be needed (11, 12). Rifampin has shown an excellent activity in bone infections and can easily penetrate tissues and biofilms, but its usage is also limited because of associated hepatotoxicity, several drug interactions, and bacterial resistance emergence (13 – 16). Few data have been reported in the literature on the minimal concentration of rifampin or ceftaroline required to inhibit the growth of the least susceptible single-step mutant (Mutant Prevention Concentrations or MPC) against MRSE (17, 18), and this requires further investigation.

New alternatives to overcome resistance of CoNs and to improve therapy of osteomyelitis are currently being explored. Ceftaroline, a cephalosporin with potent activity against methicillin-resistant staphylococci approved by the US Food and Drug Administration (FDA) in 2010 and by the European Medicines Agency (EMA) in 2012, demonstrated high clinical success rates in treating MRSA osteomyelitis in some case reports and in the CAPTURE study experience (19 – 21). In addition to its *in vitro* activity against *Staphylococcus epidermidis* including MRSE strains, ceftaroline also recently demonstrated its safe and significant efficacy when used in monotherapy in a preclinical model of MRSE osteomyelitis and in an experimental model of foreign-body MRSE infection (22, 23). However, little is known about the penetration of ceftaroline into the bone in the context of MRSE osteomyelitis and on its efficacy when used in combination with rifampin.

In this study we investigated (i) the pharmacokinetics of ceftaroline in plasma and bones in both healthy and MRSE-infected rats and (ii) the efficacy of ceftaroline alone or combined with rifampin in this rat model of MRSE-induced osteomyelitis.

## RESULTS

MPC for rifampin was >64 µg/mL against the MRSE isolate when the highest inoculum (6 × 10^9^ CFU) was plated. This MPC value decreased as the bacterial load tested was lower, confirming that the mutation frequency for rifampin is increased using a large inocula. The mean mutation frequency was 3 × 10^−8^ for rifampin (200 colonies out of 6 × 10^9^ CFU on all concentration plates from 0.015 to 64 µg/mL) in these experimental conditions.

MPC for ceftaroline was 2 µg/mL against the MRSE isolate when the highest inoculum (10^10^ CFU) was plated. This MPC value decreased to 1 µg/mL when 10^8^ CFU were plated. The mean mutation frequency was 3 × 10^−10^ for ceftaroline (three colonies out of 10^10^ CFU on the 1 µg/mL concentration plate) in these experimental conditions.

**Fig 1:**
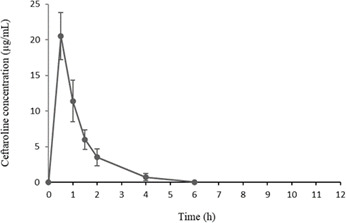
Ceftaroline mean plasma concentrations after a single 20 mg/kg intraperitoneal dose in healthy rats. The C_max_ was 20.5 µg/mL at 0.5 h, and the AUC_0-24_ using one dose every 12 h was 49.5 µg.h/mL.

Mean ceftaroline plasma concentrations versus time after a single 20 mg/kg intraperitoneal dose of ceftaroline in healthy, uninfected rats are shown in Fig. 1. Based on this pharmacokinetic profile, the maximum concentration (C_max_) was estimated to be 20.5 µg/mL on average (15.3–25.7) after 30 min (T_max_). After 12 h post-injection, ceftaroline was eliminated from the blood compartment, and the area under the concentration-time curve from 0 to 24 h (AUC_0–24_) was 49.5 µg.h/mL (considering a twice daily regimen was applied). Thus, the C_max_ achieved in the rat was similar to the C_max_ reported in humans (20.8 µg/mL on average following a 600 mg/12 h administration)(24) but the AUC in the rat was approximately half the AUC obtained in humans (97.5 µg.h/mL on average), and the % T >MIC was, therefore, about 40% in the rat, based on an MIC of 0.5 µg/mL for the MRSE strain used in this study (25).

Mean ceftaroline concentrations were also evaluated in bones of both uninfected and infected rats to evaluate the impact of the infection on the bone tissue penetration of ceftaroline at 30 min and 11 h after injection. Only results obtained at 30 min are presented in Table 1 as ceftaroline was not detectable in the bone at 11 h post-injection regardless of the group considered.

**TABLE 1:** Penetration rates of ceftaroline into the bone (compared to plasma levels) of uninfected or MRSE-infected rats receiving 1 or 14 treatment days of ceftaroline administered intraperitoneally at 20 mg/kg/12 h^*c*^

Animal type	Ceftaroline concentration at 30 min post-injection		Bone penetration ratio (%)
	Plasma (µg/mL)	Bone tissue (µg/g)	
Uninfected animals D7 (*n* = 6)	21.62 ± 2.09	0.71 ± 0.27	3.3%
Uninfected animals D21 (*n* = 6)	20.13 ± 1.93	0.87 ± 0.08	4.3%
Infected animals D7 (*n* = 6)	23.44 ± 2.90	2.45 ± 0.49	10.5% ^*a*^
Infected animals D21 (*n* = 6)	17.41 ± 0.82	1.52 ± 0.18	8.7%^*b*^

^*a*^ **** (infected animals D7 versus uninfected animals D7).

^*b*^ * (infected animals D21 versus uninfected animals D21).

^*c*^ Samples were collected 30 min after the injection. Results are shown as mean ± SD (values obtained for three animals per group, *n* = 6 tibias). D7: Start of therapy. Quantitative variables were compared using analysis of variance and a post-hoc analysis using Bonferroni’s test. *P* < 0.05 was considered significant * *P* < 0.05, **P < 0.01, *** *P* < 0.001, **** *P* < 0.0001.

In uninfected tibias, ceftaroline reached a peak concentration (C_max_) of 0.71 ± 0.27 µg/g on average on treatment day 1 (meaning at D7). In tibias of infected animals, the C_max_ was 2.45 ± 0.49 µg/g on average. The penetration ratio (bone versus plasma) of ceftaroline was thus significantly higher in infected bones (10.5%) than in uninfected bones (3.3%) at D7 (*P* < 0.0001) and to a lesser extent at D21 (*P* < 0.05). Of note, there was no significant impact of the time in a given group (uninfected or infected) on the ceftaroline penetration ratio. In order to assess a possible accumulation of ceftaroline after repeated administrations over 14 days of treatment, the assays were carried out in the same way in uninfected or infected animals at D21. No accumulation was observed regardless of the group and a two times higher penetration ratio was recorded in infected bones (8.7%) compared to uninfected bones (4.3%), suggesting that the infection could enhance the proportion of ceftaroline that reaches the bone compartment. However, these penetration data were obtained from a single timepoint and will have to be confirmed over several timepoints.

**Fig 2:**
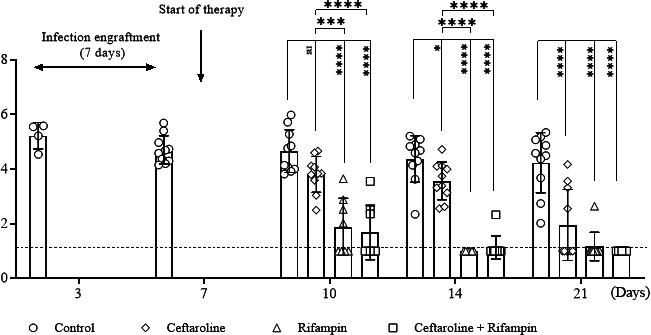
Results of quantitative MRSE bone cultures (log10 CFU per gram of bone) from rats receiving vehicle, ceftaroline alone, rifampin alone, or the combined therapy. Individual tibia results are shown for each study group (*n* = number of tibias, 2 tibias per animal). Results are expressed as mean ± SD. D7: Start of therapy. Quantitative variables were compared using an analysis of variance and a post-hoc analysis using Bonferroni’s test. (*P < 0.05, **P < 0.01, ***P < 0.001, ****P < 0.0001, versus controls).

Bacterial loads in bone cultures for each rat are shown in Fig. 2. A total of 67 rats were used for the study; four of them died during anesthesia meaning a total of 63 rats (126 tibias) were included in the analysis: two control animals at D3 (*n* = 4 tibias), then three to five animals (*n* = 6 to 10 tibias) per group from D7 to D21. Overall, no mortality was recorded after bacterial challenge, and all animals recovered their mobility 12 h after the surgery.

At the start of therapy (D7), the mean bacterial count ± standard deviation in the control group was 4.7 ± 0.5 log_10_ CFU/g. The bacterial load in this group slightly decreased between D3 (5.2 ± 0.5 log_10_ CFU/g) and D7 but was stable until the end of the experiment at D21 (4.2 ± 1.1 log_10_ CFU/g). None of the control animals recovered spontaneously from their infection during the observation period of 21 days.

After a 3-day treatment period (D10 post-infection), bacterial load in tibias of animals receiving rifampin alone or combined with ceftaroline decreased significantly (1.89 ± 1.0 log_10_ CFU/g, *P* < 0.0001 and 1.68 ± 1.0 log_10_ CFU/g, *P* < 0.0001*,* respectively), compared with the saline-treated control group. The bacterial load in tibias from animals receiving ceftaroline alone also decreased but in a non-significant manner (3.8 ± 0.6 log_10_ CFU/g).

After a 7-day treatment period (D14 post-infection) using rifampin alone or combined with ceftaroline, a complete sterilization of the tibias in almost all animals was recorded (only one tibia from an animal receiving the rifampin/ceftaroline combination had a bacterial load of 1.1 ± 0.4 log_10_ CFU/g). A treatment using ceftaroline alone significantly decreased the bacterial load in tibias (3.6 ± 0.7 log_10_ CFU/g versus 4.4 ± 0.8 log_10_ CFU/g in control animals, *P* < 0.05), but none of the tibias was completely sterilized.

Finally, after a 14-day treatment period (D21 post-infection), no MRSE was recovered from the tibias of animals receiving rifampin alone or combined, except for a single tibia from a rat receiving rifampin alone that remained positive at a very low level (1.2 ± 0.5 log_10_ CFU/g of bone; 90% of sterilization). The group receiving ceftaroline alone had a higher bacterial load recovered from the bone, but this bacterial load remained very low and close to the detection threshold (2.0 ± 1.3 log_10_ CFU/g of bone; 60% of sterilization).

Emergence of resistance was not observed in any of the treated groups when plating the pulverized bone of animals onto agar plates containing two and four times the MIC of the tested strain.

## DISCUSSION

Staphylococcal osteomyelitis represents an important clinical burden because of complicated and prolonged treatment associated with high morbidity rates and hospital costs. Here, the efficacy of ceftaroline alone or associated with rifampin was evaluated in a rat model of methicillin-resistant *S. epidermidis* (MRSE) osteomyelitis without implant. In this study, a ceftaroline monotherapy demonstrated significant efficacy against MRSE after 7 days of treatment (1 log reduction on average) until 14 days of treatment (2 log reduction on average). These results provide evidence that a 600 mg/12 h ceftaroline regimen would be likely to achieve a successful bone antibacterial activity in humans as the exposure is enhanced compared to rats and are consistent with the reported clinical success rates observed in the CAPTURE study, enrolling patients with osteomyelitis and treated with ceftaroline fosamil [95% of success in patients infected with CoNS (19)].

A monotherapy using rifampin was more rapidly bactericidal in this MRSE model as a >2 log reduction was already observed from the third day of treatment; all animals receiving rifampin alone had sterile bone culture after 7 days of treatment. A similar outcome was observed in terms of antibacterial efficacy in animals receiving the ceftaroline-plus-rifampin combination therapy as a 3-log reduction was recorded only after 3 days of treatment. However, considering the high bacterial reduction obtained in animals receiving rifampin alone, a potential synergy by adding ceftaroline was not observed.

While rifampin monotherapy is not recommended in clinical practice, the association with ceftaroline could represent a valuable option to prevent the selection of resistance. However, in our study, no resistant mutants were detected in animals receiving rifampin alone or combined with ceftaroline over the 14-day treatment period. The absence of resistant mutants is consistent with the inoculum size at the initiation of the treatment [around 5 log CFU/g, which can be similar to clinical findings (4)] and the rate of sterilization achieved after a 2 weeks-period of therapy. In comparison, the MPC values obtained for rifampin were similar to the few values published in the literature (17) and above the attainable maximum serum concentrations at the currently recommended doses, only when a high inoculum was tested. The mutation frequency for rifampin was approximately 3 × 10^−8^, similar to other data reported in the literature (10^–6^–10^–8^) against planktonic *S. aureus* (26). The bacterial density in our model is probably not large enough to select mutant bacterial populations. In parallel, we deliberately chose a higher dosage of rifampin (25 mg/kg instead of 10 mg/kg usually applied in rodents to reproduce the human exposure). Because of fears of toxicity, rifampin was historically introduced at an oral daily dose of 600 mg in the treatment of tuberculosis (equivalent to a 10–12 mg/kg dosage), but several lines of evidence suggest that higher rifampin doses, if safe, could help to achieve the PKPD targets and avoid the emergence of resistance (27). This hypothesis could also explain why no resistant mutant was recovered in animals receiving a rifampin monotherapy in this experimental study.

The tested MRSE isolate had a low MPC value for ceftaroline (2 µg/mL) compared to rifampin and consequently a very narrow Mutant Selection Window (within 0.5–2 µg/mL), indicating that ceftaroline exhibits a better ability to prevent the emergence of mutants. As far as we know, few data on the MPC values for ceftaroline have been reported in the literature, with the exception of the study published by Gostev et al. on *S. aureus* strains (18). Further investigations on the determination of MPC for ceftaroline on a larger panel of *S. epidermidis* strains are therefore required.

Furthermore, additional studies evaluating the utility of the combination ceftaroline-rifampin on the limitation of resistance could be carried out in other MRSE models, in particular those involving an implant associated biofilm and/or a higher bacterial load.

Ceftaroline (alone or in combination with rifampin) was clinically well tolerated by animals throughout the whole duration of treatment (no fur loss, no diarrhea or soft feces, and a 10% weight gain from baseline); these observations were already demonstrated by several authors in refractory MRSA infections such as bacteriemia or endocarditis where ceftaroline was well tolerated and effective (23, 28 – 30).

The serum pharmacokinetic profile observed in the rat after a single intraperitoneal administration of ceftaroline (20 mg/kg) was similar to the PK data obtained in the rat after an intramuscular injection (31), but the total exposure (AUC_0-24_) was 50% lower than the one obtained in human. However, this 20 mg/kg/12 h dosage was kept because it had been used during the previous study, rifampin in the combination group was administered every 12 h (and too many injections for a long period of time were not welcomed for ethical reasons), and lastly, a synergistic effect was looked for when rifampin was added to the ceftaroline therapy.

Using this 20 mg/kg/12 h ceftaroline regimen, the serum %T >MIC (which is the key PK/PD efficacy index for β‐lactams) achieved in the rat was about 40% (expressed as the total drug concentration), considering an MIC of 0.5 µg/mL for the strain used in this study. This %T >MIC is half of the value that would have been obtained in a human receiving a 600 mg/12 h regimen (%T >MIC = 80% on average, based on an MIC of 0.5 µg/mL). The ceftaroline protein binding was not performed in this study but is expected to be around 30–40%, assuming a similar protein binding to mice or rabbits (32, 33). Thus, a total T >MIC of 40% would be equal to a free T >MIC of 26% on average in rats. The most commonly average %fT >MIC targets reported for ceftaroline against *S. aureus* for stasis, 1-log_10_ kill, and 2-log_10_ kill are 27%, 31%, and 35%, respectively, but these values relate to *S. aureus* infections (and not *S. epidermidis* infections) and to acute infections (and not subacute/chronic infections) (34). Also, numerous *in vitro* and *in vivo* models and clinical trials have suggested that the magnitude of the %T >MIC predictive of cephalosporin efficacy ranges from 25% to 70%, depending upon the defined therapeutic endpoints. For example, we previously reported that a free %T >MIC as low as 25 was associated with a complete eradication of penicillin-resistant *S. pneumoniae* in a rabbit pneumonia model (35).

In the case of this specific MRSE osteomyelitis model, the exposure to ceftaroline, even lower than in humans, was effective on strain having an MIC of 0.5 µg/mL. Additional strains having higher MICs should be further investigated in this model.

The serum/bone ceftaroline concentration ratio was also evaluated in both noninfected and infected animals and after a short or a long treatment duration. The bone concentrations were measured on the whole tibia of animals, and no distinction was made between the cortical bone and the medullary bone. Overall, no accumulation was observed as the penetration ratios in uninfected bones were 3.3% and 4.3% in the short and in the extended duration dosing group, respectively. In comparison, a more than twice higher penetration ratio was recorded in infected bones (8.7%–10.5%) compared to uninfected bones at least at the T_max_ (30 min), suggesting that the infection could enhance the proportion of ceftaroline that reaches the bone compartment. Interestingly, the ceftaroline penetration ratio slightly decreased in infected bones over time (10.5% at D7 versus 8.7% at D21), which could be coherent with the regression of the infection. These preclinical findings are valuable as such data are difficult to obtain in clinical trials because of recruitment issues, heterogeneity of clinical situations, sampling procedures, and treatment regimens (36). However, they should be interpreted with caution as only C_max_ and C_min_ parameters were investigated in this experimental study; thus, the entire PK curve (and consequently the overall drug exposure) in bone could not be described in detail. In particular, the percentage of time above MIC (%T >MIC), which is the key pharmacokinetic/pharmacodynamic (PK/PD) efficacy index for ceftaroline, could not be estimated and further investigations are needed to assess the penetration ratio of ceftaroline at several timepoints. However, the CFU data obtained from animals receiving ceftaroline alone provide evidence that ceftaroline distributes in the bone of rats at concentrations that are low (1.52 to 2.45 µg/g on average) but sufficient regarding the MIC of the tested strain (0.5 µg/mL) to induce a significant effect for the tested period. Similar observations were also reported by Jacqueline et al. in an MRSA acute osteomyelitis model in rabbits (37).

The bone PK investigations were not performed for rifampin in this study as several data in the literature indicate its excellent penetration into bone tissues (cortical and medullar) at high levels (10). The bone PK of ceftaroline when co-administered with rifampin could also be further explored to assess whether there is an impact on the PK (plasma and bone) of each of the antibiotics when they are co-administered. However, ceftaroline is not metabolized by CYP enzymes; therefore, co-administration with CYP inducers or inhibitors is unlikely to influence the pharmacokinetics of ceftaroline (38).

There are some limitations to this study; the first one being that this model is rather a subacute model of osteomyelitis not involving any implant, which is probably easier to treat than chronic osteomyelitis. Thus, this treatment should be tested in other *S. epidermidis* models, such as those involving a foreign body and associated biofilm formation. Here, only one strain of MRSE with very low MICs for rifampin and ceftaroline was tested, meaning that this combination therapy deserves further evaluation on other strains as a potential treatment option for MRSE osteomyelitis. Finally, significant differences in pharmacokinetics can be observed between cancellous and cortical bone, suggesting that bone may not be considered as one compartment. Here, the bone PK was performed on pulverized whole tibias due to their small size; but future studies should focus on validating the applicability of microtechniques for assessment of antimicrobial bone pharmacokinetics.

In conclusion, ceftaroline monotherapy demonstrated a significant but slower bactericidal activity than a rifampin monotherapy in this rat sub-acute MRSE osteomyelitis model; these findings are consistent with the lower exposition to ceftaroline, observed in rats compared with the humans. Rifampin monotherapy reduced the bacterial load very rapidly, improving the bacterial outcome of the infection and preventing the demonstration of potential synergy between ceftaroline and rifampin. Although no resistant mutant was recovered after treatment with rifampicin (alone or in combination) in this model, ceftaroline and rifampin combination therapy could represent a valuable therapeutic option for human MRSE osteomyelitis and deserves further preclinical and clinical evaluation.

## MATERIALS AND METHODS

### Bacterial strain, growth conditions, and antibiotics

The clinical methicillin-resistant *S. epidermidis* 9120486910–1 isolate, originally recovered from a patient suffering from a diabetic foot associated monomicrobial osteomyelitis, was studied (Assistance Publique des Hôpitaux de Paris). Bacterial stocks were kept at −80°C in cryobeads (bioMérieux, Marcy l’Etoile, France). MICs were performed in triplicate by broth microdilution using cation adjusted Mueller-Hinton (MH) broth (Becton Dickinson, France) and according to EUCAST guidelines. This strain was resistant to methicillin but susceptible to ceftaroline (MIC = 0.5 µg/mL) and rifampin (MIC = 0.015 µg/mL). It was grown either on Chapman agar plates or Mueller-Hinton agar plates or in Brain Heart Infusion liquid medium (bioMérieux, Marcy l’Etoile, France).

For *in vitro* experiments, ceftaroline dihydrochloride was provided by Pfizer (Milwaukee, USA), and rifampin was purchased from Sigma Aldrich (Saint Quentin Fallavier, France). For *in vivo* studies, commercial formulations Zinforo (600 mg Pfizer) and Rifadine (600 mg Sanofi-Aventis) were reconstituted in 0.9% serum saline (Fresenius Kabi).

*In vitro* rifampin and ceftaroline MPC (Mutant Prevention Concentration) determination was performed according to the protocol described by Zhaoand Drlica (39). MH plates containing rifampin or ceftaroline at various concentrations (including the MIC value as the lowest concentration to 64 µg/mL as the highest concentration) were inoculated with 100 µL of a 6 × 10^10^ CFU/mL to 10^11^ CFU/mL bacterial suspension (8 × 10^9^ CFU/mL to 10^10^ CFU/mL per plate) or of serial successive 1/10 dilutions (to assess the impact of inoculum size on MPC values). MPC was recorded as the lowest antibiotic concentration that prevented bacterial colony formation after 72 h of incubation at 37°C in ambient air. In addition, an enumeration of mutant colonies was performed to assess the mutation frequency.

### Preparation of bacterial inocula for *in vivo* experiments

Based on preliminary experiments (data not shown), an infective dose of 5.2 × 10^9^ CFU/tibia was selected to induce a stable osteomyelitis for 3 weeks of infection. Thus, before each animal experiment, the staphylococcal strain from one frozen aliquot was freshly cultured on Chapman agar plate (bioMérieux, Marcy l’Etoile, France) and incubated aerobically for 48 h at 37°C. One colony was inoculated into 10 mL of Brain Heart Infusion (bioMérieux, Marcy l’Etoile, France) and incubated for 6 h at 37°C with agitation. The resulting bacterial suspension was then spread onto 10 Mueller Hinton agar plates (bioMérieux, Marcy l’Etoile, France) and incubated for 18 h at 37°C. The bacterial layer was scraped and homogenized in 10 mL of sterile serum saline containing glass beads to obtain a 10^11^ CFU/mL bacterial suspension. Viable bacterial counts were determined by plating successive dilution cultures on agar plates.

### Animals and ethical aspects

The experimental osteomyelitis model was established in immunocompetent male Wistar rats weighing 250 to 300 g, as described by O’Reilly and Mader (40). Animals were housed three per cage due to their gregarious lifestyle, with access to water and food *ad libitum*, according to the current recommendations of the European Institute of Health EU Directive 86/609. The experimental protocol was approved by the local ethics committee for animal experimentation (APAFIS #32401).

### Experimental osteomyelitis model

**Fig 3:**
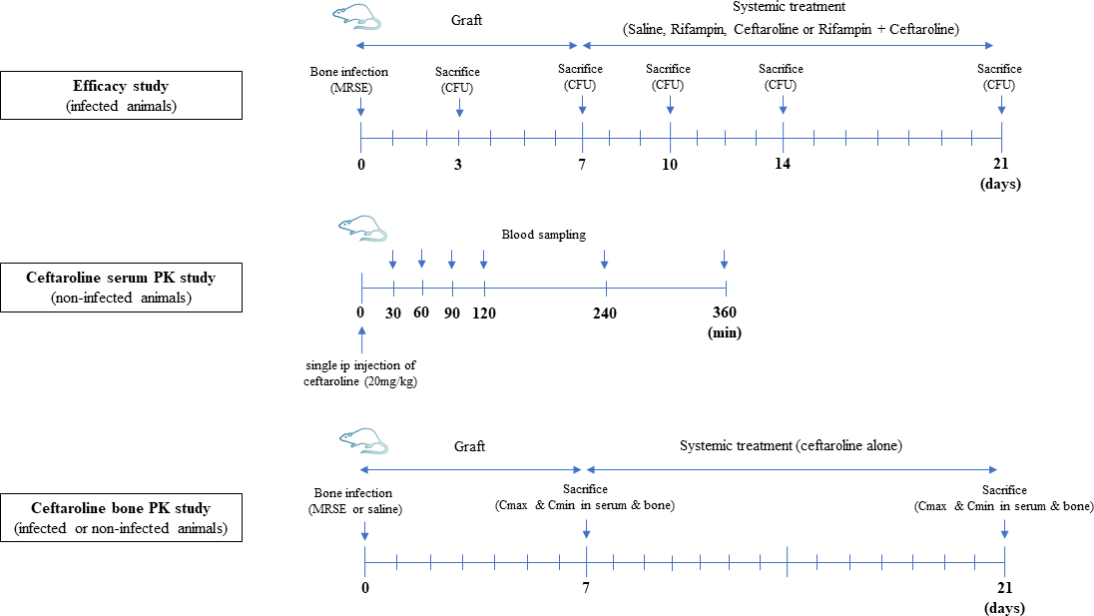
Experimental design summary.

The entire experimental design summary is shown in Fig. 3. The tibia osteomyelitis rat model was established as previously described by Albac et al. (22). Animals were anaesthetized by intraperitoneal administration of ketamine (90 mg/kg of body weight, Virbac) and xylazine (10 mg/kg of body weight, Bayer). Legs were shaved and disinfected three times with polyvinylpyrrolidone-iodine (Betadine). The anterior tibial metaphysic of each leg was surgically exposed, and a 1.5 mm hole was drilled through the cortex into the medullary cavity using a high-speed drill with a 0.5 mm diameter bit. 50 µL of the inoculum (1 × 10^11^ CFU per mL) was slowly inoculated into the bone. No adjuvant was used. The hole was covered with sterile dental gypsum. The fascia and skin were closed with sutures (Ethicon, 5–0), and the wound was daily disinfected for 3 days following the surgical procedure to avoid contamination. Buprenorphine (MedVet) was administered subcutaneously for analgesia (0.05 mg/kg). Animals were monitored on a daily basis. In the first days, food access was facilitated by placing croquettes directly into the cage.

### Animal treatment

After 7 days of infection (graft period), antibiotic treatment was initiated (at D7) with animals being treated intraperitoneally up to D21 (either for 3, 7, or 14 days of the treatment period). To confirm the bacterial load in the bone at treatment initiation, control animals were culled after 7 days of infection and quantitative bone cultures were performed. Animals were randomly assigned to one of the four study arms: no treatment (0.9% saline serum), ceftaroline alone (20 mg/kg/12 h), rifampin alone (25 mg/kg/12 h), ceftaroline (20 mg/kg/12 h), and rifampin (25 mg/kg/12 h). Ceftaroline dose used in our published rat osteomyelitis model was administered, and rifampin dose was selected on the basis of data published in a rat model of MRSA osteomyelitis (22, 41)).

Twelve hours after the end of the therapy to avoid any carry over effect, animals were intraperitoneally anaesthetized (using the mixture of ketamine plus xylazine) and culled by an intracardiac overdose of pentobarbital (Euthasol, Virbac).

### Evaluation of infection

Right and left tibias of each animal were dislocated and stored at –80°C until they were crushed in liquid nitrogen using a cryo-crusher (Delta Labo, France). The pulverized bone was weighed, and an amount was resuspended in 1 mL of sterile PBS, vortexed, and quantitative culture was performed by plating serial dilutions of this sample on Chapman agar plates. Results were expressed in log_10_ CFU/g of bone. If the identification of colonies was uncertain on Chapman agar plates, an identification using a MALDI-TOF was performed (Ultra Flex Speed, Bruker Daltonics). Untreated but infected animals were used as controls. Surviving bacteria in bone were assessed on D10, D14, and D21 after infection (meaning D3, D7, and D14 after the initiation of the treatment) depending on the different therapies. For statistical comparisons of the differences between bone bacterial densities, culture-negative samples were considered to contain 1 log_10_ CFU/g.

### Statistical analysis

Statistical analysis was performed using GraphPad Prism version 7.00 software. Quantitative variables were compared using a Mann-Whitney U test or analysis of variance and post-hoc analysis using Bonferroni’s test. *P* < 0.05 or less was considered significant for all tests performed.

### Pharmacokinetic study

*Plasma pharmacokinetics*: ceftaroline plasma concentrations in uninfected or infected rats were determined after the intraperitoneal administration of 20 mg/kg of ceftaroline. Blood samples of approximately 0.5 mL were collected from animals through the tail vein at 30-, 60-, 90-, 120–240-, and 360 min post-injection for analysis. These animals were kept alive for 1 week (wash out period and time for recovery) and were reused for the PK tissue study (non-infected group).

*Bone pharmacokinetics:* Ceftaroline concentrations following a 20 mg/kg dosing were evaluated in bone tissue of both uninfected and infected animals (*n* = 12 per group) to assess the impact of the infection. For both groups, this evaluation was performed on the first day of treatment (D7) and on the last day of treatment (D21) to assess any accumulation of ceftaroline into the bone after 14 consecutive days of treatment. Bones were collected 30 min and 11 h post-injection of ceftaroline at 20 mg/kg (corresponding to the C_max_ and C_min_ of ceftaroline, respectively), then cautiously wiped on sterile compresses to avoid blood contamination, and finally crushed in liquid nitrogen using a cryo-crusher as described above and stored at −80°C until analysis. Blood samples were individually collected at the same time as the bones.

### Ceftaroline assay

The plasma and bone concentrations of ceftaroline were assessed using an Ultra-Performance Liquid Chromatography–Tandem Mass Spectrometry (UPLC-MS/MS) method (INSERM U1070, Université de Poitiers).

For both matrixes, a Solid Phase Extraction (SPE) was used using the cartridge SPE IST EVOLUTE, 25 mg, 1 mL, ABN, Biotage. The equipment was composed of a quadrupole tandem mass spectrometer (API QUATTRO micro, Waters) and HPLC (alliance 2695) with a data acquisition station: MassLynx version 4.1.

The analytical column was an Xterra C18 column (5 µm 2.1*50 mm) (Waters). Internal Standard (IS) is the (13C, 2H3) ceftaroline. The mobile phase consisted of a mixture of 10 mM ammonium formate contained in water, acetonitrile and water (16.4%/1/%, 65.6%, vol/vol/vol) using an isocratic mode at 25°C with a flow as 0.3 mL/min. For the MS/MS detection, the electrospray ionization in positive mode was applied. Mass spectra were acquired by multiple reaction monitoring. The specific transition used for quantification is 605– >208.1 m/z and 609– >212.1 for ceftaroline and IS, respectively. The retention time was about 1 min for both compounds.

The range concentration was between 10 and 10,000 ng/100 mg for bone samples and 10/10,000 ng/mL for plasma samples.
